# Battle of the CH motions: aliphatic versus aromatic contributions to astronomical PAH emission and exploration of the aliphatic, aromatic, and ethynyl CH stretches

**DOI:** 10.1093/mnras/stae2588

**Published:** 2024-11-15

**Authors:** Vincent J Esposito, Salma Bejaoui, Brant E Billinghurst, Christiaan Boersma, Ryan C Fortenberry, Farid Salama

**Affiliations:** NASA Ames Research Center, Astrophysics Branch, Moffett Field, CA 94035, USA; NASA Ames Research Center, Astrophysics Branch, Moffett Field, CA 94035, USA; Canadian Light Source Inc., 44 Innovation Boulevard, Saskatoon, 11 Saskatchewan S7N 2V3, Canada; NASA Ames Research Center, Astrophysics Branch, Moffett Field, CA 94035, USA; Department of Chemistry & Biochemistry, University of Mississippi, University, MS 38677, USA; NASA Ames Research Center, Astrophysics Branch, Moffett Field, CA 94035, USA

**Keywords:** astrochemistry, molecular data, methods: laboratory: molecular, techniques: spectroscopic, ISM: molecules

## Abstract

Strong anharmonic coupling between vibrational states in polycyclic aromatic hydrocarbons (PAH) produces highly mixed vibrational transitions that challenge the current understanding of the nature of the astronomical mid-infrared PAH emission bands. Traditionally, PAH emission bands have been characterized as either aromatic or aliphatic, and this assignment is used to determine the fraction of aliphatic carbon in astronomical sources. In reality, each of the transitions previously utilized for such an attribution is highly mixed with contributions from both aliphatic and aromatic CH motions as well as non-CH motions such as CC stretches. High-resolution gas-phase IR absorption measurements of the spectra of the aromatic molecules indene and 2-ethynyltoluene at the Canadian Light Source combined with high-level anharmonic quantum chemical computations reveal the complex nature of these transitions, implying that the use of these features as a marker for the aliphatic fraction in astronomical sources is not uniquely true or actually predictive. Further, the presence of aliphatic, aromatic, and ethynyl CH groups in 2-ethynyltoluene provides an internally consistent opportunity to simultaneously study the spectroscopy of all three astronomically important groups. Finally, this study makes an explicit connection between fundamental quantum mechanical principles and macroscopic astronomical chemical physics, an important link necessary to untangle the lifecycle of stellar and planetary systems.

## 1 INTRODUCTION

Aromatic molecules have long been postulated to be present in various astronomical environments based on their ubiquitous, distinct, and intense infrared (IR) signature that spans the 3–20 µm wavelength region. These bands are commonly termed the aromatic IR bands (AIBs); see Tielens (2008) for a review. Besides benzene, which was first detected through its IR absorption towards the protoplanetary nebula CRL 618, (Cernicharo et al. 2001) there was no specific assignment of aromatic molecules for decades due to the lack of molecular specificity of IR transitions (although the non-aromatic buckminsterfullerene (C$_{60}$) was detected via its vibronic transitions in the near-IR; Cami et al. 2010). That is, IR observations are mostly representative of functional groups and, thus, usually do not allow for the detection of an individual, complex molecule. Though recently, rotational spectroscopy has been used to search for specific aromatic molecules with a strong permanent dipole moment via radio observations. In 2018, the radio-detection of benzonitrile in the Taurus Molecular Cloud (TMC-1) confirmed the existence of a specific, unique aromatic molecule in space and the presumed existence of aromatic molecules as a class in general (McGuire et al. 2018). Since then, the cyano-substituted polycyclic aromatic hydrocarbons (PAHs) cyanonaphthalene (McGuire et al. 2021) and 2-cyanoindene (Sita et al. 2022) have also been detected. The highly polar nitrile functional group on these ‘exotic’ PAH species enables their detection via radio astronomy, but their abundance ratios compared to that of their unsubstituted counterpart remain unclear. This makes the detection of indene (Burkhardt et al. 2021; Cernicharo et al. 2021), the first and to-date only pure astronomically known PAH as detected in TMC-1, all the more vital for gauging the abundances of PAHs.

The carbon-hydrogen chemical bond is one of the simplest yet most prolific bonds throughout nature, and PAHs are no exception. The CH stretches of PAHs have been a marker of their astronomical emission since the onset of the astronomical PAH model with ground-based observations and opened wide with the launch of space-based IR telescopes such as *ISO, Akari*, and now *JWST* (Tielens 2008; Li 2020; Boersma et al. 2023; Chown et al. 2024; Esposito et al. 2024f). Even with their ubiquity and notability, CH stretches in PAHs come in many different and distinct flavours. Aliphatic CH stretches arise from hydrogen attached to an sp$^3$ hybridized carbon that can be either positioned within a ring or on an alkyl chain; they typically have a frequency in the range of 2900–2850 cm^$^{-1}$^ (3.4–3.5 µm). Aromatic CH stretches originate from the C–H bond on an aromatic ring and have a slightly higher frequency of around 3030 cm^$^{-1}$^ (3.3 µm). Ethynyl CH stretches, the most intense of the group, arise from the hydrogen atom at the end of the carbon–carbon triple bond of an acetylide group bonded to the PAH and occur around 3300 cm^$^{-1}$^ (3.0 µm). This progression in frequency provides an excellent marker for the types of hydrocarbons being observed. That said, so far, only a single ethynyl-substituted aromatic molecule has been detected in space: ethynylbenzene (Loru et al. 2023). Such a lack of detection can, at least in part, be attributed to insufficient reference data being available for comparison to astronomical observations, implying that more spectroscopic work on these molecules must be undertaken in order for data from *JWST* to be better interpreted.

In astronomical IR spectra, the 3.3 µm band thought to arise from the aromatic CH stretches is one of the major emission bands of the AIBs (Tielens 2008). The notable features at 3.3, 6.2, 7.7, 11.2, and 12.7 µm are commonly all attributed to originate from different stretching and bending modes in PAHs. A series of bands in the 3.4–3.5 µm range are currently posited to belong only to emission from aliphatic CH stretches of PAHs (Tielens 2008; Li 2020; Yang & Li 2023b). Many ‘minor’ bands are interspersed between these bands or show up as satellite features, some of which remain currently unassigned.

The relative integrated emission intensity of the aromatic and aliphatic CH bands provides insight into the formation and destruction of PAHs (Hudgins, Bauschlicher & Sandford 2004; Peeters et al. 2004; Sloan et al. 2007; Yang et al. 2013; Mackie et al. 2018; Shannon & Boersma 2019; Allamandola et al. 2021; Yang & Li 2023a, b). The current state of the PAH model predicts that aromatic molecules such as PAHs are the main carriers of the AIBs (Chiar et al. 2013), with a median PAH aliphatic/aromatic fraction (commonly derived from the *I*$_{3.4}$/*I*$_{3.3}$ intensity ratio) of 5.4 per cent (Yang & Li 2023b). This fraction could be used to decipher the carriers of the main AIBs. Currently, the 3.4–3.5 µm bands are attributed solely to aliphatic CH stretches; however, these features may have other contributors, such as overtones and combination bands of lower frequency modes and anharmonic effects of aromatic CH stretches (Allamandola et al. 2021). If indeed so, the methods used to determine the aliphatic fraction need to be modified. Features at 6.85 and 7.25 µm, attributed to aliphatic C–H deformation modes, can be used to probe this fraction as well (Yang et al. 2016). JWST with its 0.6–28 µm wavelength coverage has the capabilities to provide enough resolution to, for the first time, precisely analyse the spectral features and allow for truly examining this ratio considering all three features. In order for this to transpire, though, the right molecule(s) must be selected to examine these spectral features.

Indene (C$_9$H$_8$) is postulated to be a key intermediate in the bottom-up growth mechanism for PAHs in the gas phase. Its role has been explored extensively using quantum chemistry and experimental pyrolysis approaches (Lu & Mulholland 2001; Fascella et al. 2004; Ajaz et al. 2014; Wentrup, Winter & Kvaskoff 2015; Mebel, Landera & Kaiser 2017; Jin et al. 2019). In one experiment (Ajaz et al. 2014), pyrolysis of 2-ethynyltoluene (C$_9$H$_8$), an isomer of indene, led to the formation of indene as well as larger PAHs such as chrysene (C$_{18}$H$_{12}$) and benzo[a]anthracene (C$_{18}$H$_{12}$). This study implicates 2-ethynyltoluene, and alkylated PAHs in general (Gavilan Marin et al. 2020), as a key precursor in the growth of PAHs in high-energy regions, as well as a possible proxy for the destruction of indene. Interestingly, 2-ethynyltoluene is the methyl-substituted daughter of the astronomically detected ethynylbenzene (C$_8$H$_{6}$), and contains all three types of CH bonds (aromatic, aliphatic, and ethynyl). The role that 2-ethynyltoluene plays in the formation of indene or even larger PAHs has yet to be completely captured. In either case of 2-ethynyltoluene or indene, the full set of 1–20 µm spectral features is needed to explore such chemistry *in situ* with JWST. The gas-phase optical absorption (Chu et al. 2023) and IR studies of indene in the solid phase, both in the pure form and in water ice (Mate et al. 2023), are a starting point for its spectral characterization. However, the differences between spectra observed in solid versus gas implies that further gas-phase data are necessary. While gas-phase vibronic spectra of the D$_2$ state have been obtained for the indene cation (Chalyavi et al. 2013) and the harmonic emission spectrum of indene has been simulated and discussed in an astronomical context (Li et al. 2024) there is a distinct lack of gas-phase IR absorption data on *neutral* indene, the actual molecule discovered in space.

Consequently, this paper presents, for the first time, the high-resolution gas-phase absorption spectra of neutral indene (${\rm C_9H_8}$) and its isomer 2-ethynyltoluene as captured from experiments at the Canadian Light Source. Furthermore, these spectral features are characterized by comparisons to highly accurate, anharmonic quantum chemical, theoretical computations. The theoretical results are used to explain and assign the experimental findings, and the experiments are used to benchmark the theory both for the present and also for future applications. Furthermore, the anharmonic IR cascade emission spectra for each molecule are simulated in order to provide the most-comparable benchmarks for what JWST observations of either molecule would look like. The results herein are discussed regarding their implications on laboratory and observational astrophysics as well as for potential future observations with the NIRSpec instrument aboard JWST.

## 2 METHODS

### 2.1 Experimental methods

High-resolution IR absorption spectra of indene and 2-ethynyltoluene (${\rm C_9H_8}$) are measured using a Bruker IFS 125 HR FTIR spectrometer at the far-IR beamline of the Canadian Light Source (May 2024; Winnewisser et al. 2014). Indene and 2-ethynyltoluene are supplied by Sigma-Aldrich with stated isotopic purity of 99 and 97 per cent, respectively, and used without further purification. Measurements are performed at 294.35 K in a 2-meter-long multi-pass cell with an effective path length of 72 meters. The cell is first evacuated to a pressure below 1 mtorr, then filled to 6.7 and 7.1 mtorr of indene and 2-ethynyltoluene, respectively, to limit pressure broadening. The absorption spectra are recorded in the 700–4000 cm^$^{-1}$^ range, where the optimal instrumental performance is achieved with a Globar source, KBr beam splitter, and MCT Narrow band detector. The absorption spectra are collected at maximum resolution (0.00096 cm^$^{-1}$^) and averaged over $\sim$120 scans and background subtracted from reference spectra collected with the evacuated cell.

### 2.2 Computational methods

#### 2.2.1 Anharmonic absorption spectra

The optimized geometry, harmonic frequencies, and normal modes of indene and 2-ethynyltoluene are computed with the B3LYP density functional (Becke 1993) in conjunction with the N07D basis set (Barone, Cimino & Stendardo 2008) using Gaussian 16 (Frisch et al. 2016). The geometry optimization uses an energy convergence threshold of 1$\times$ 10^$^{-12}$^ and a custom integration grid consisting of 200 radial shells and 974 angular points per shell (Maltseva et al. 2015). The N07D basis set is based on the double-$\zeta$ 6–31G(d) basis set with additional diffuse and polarization functions added that have been shown to better treat anharmonicity in large, aromatic systems such as PAHs (Barone, Biczysko & Bloino 2014; Maltseva et al. 2015; Mackie et al. 2015b).

Following optimization, a quartic force field (QFF) is computed at the same level of theory using Gaussian 16. A QFF is a fourth-order Taylor expansion of the potential energy surface around the equilibrium geometry and consists of the quadratic, cubic, and quartic normal coordinate force constants (*X*) with the following formula:


(1) \begin{eqnarray*}
V &= & \frac{1}{2}\sum _{i,j}^{3N} {\biggl (\frac{\partial ^2 V}{\partial X_i\partial X_j}\biggl)X_iX_j} \\
&&+ \frac{1}{6}\sum _{i,j,k}^{3N} {\biggl (\frac{\partial ^3 V}{\partial X_i\partial X_j\partial X_k}\biggl)X_iX_jX_k} \\
&&+ \frac{1}{24}\sum _{i,j,k,l}^{3N} {\biggl (\frac{\partial ^4 V}{\partial X_i\partial X_j\partial X_k\partial X_l}\biggl)X_iX_jX_kX_l},
\end{eqnarray*}

where the coefficient is 1/*n*! and the sum is over the 3N normal coordinates of the *n*th partial derivative with respect to each normal coordinate. A linear transformation is used to transform the QFF into Cartesian coordinates (Mackie et al. 2015a).

Anharmonic frequencies are then computed with second-order rovibrational perturbation theory (VPT2) (Mills 1972; Watson 1977; Fortenberry & Lee 2019; Franke, Stanton & Douberly 2021; Fortenberry & Lee 2022) using a modified version of the spectro software (Gaw et al. 1991). spectro implements a polyad resonance matrix approach to VPT2 (Martin et al. 1995; Martin & Taylor 1997; Mackie et al. 2015b). These matrices allow for the advanced treatment of resonance interactions through separating states by frequency and symmetry. When the density of vibrational states is high, which is often the case for PAHs, each state can participate in multiple resonances at the same time. This is called ‘resonance chaining’. The resonance polyad matrices within spectro allow for simultaneous treatment of these resonance chains as well as accounting for the redistribution of intensity that stems from the anharmonic coupling, which are difficult to describe with standard VPT2 implementations (Mackie et al. 2015b; Esposito et al. 2024f). States with a separation of less than or equal to 200 cm^$^{-1}$^ are included in the resonance polyads (Mackie et al. 2015b; Esposito et al. 2024c) and vibrational modes with frequencies below 300 cm^$^{-1}$^ are excluded from the VPT2 treatment due to issues in accurately describing their potential surface with anharmonic methods (Westbrook et al. 2021; Firth, Bell & Fortenberry 2024; Watrous, Davis & Fortenberry 2024; Esposito et al. 2024a, b).

#### 2.2.2 Anharmonic emission spectra

In space, PAHs absorb UV photons causing an electronic excitation. Internal conversion to the electronic ground state and subsequent internal vibrational energy redistribution (IVR) populates the vibrational modes of the PAHs (Felker & Zewail 1984). Following this, radiative relaxation via the emission of IR photons occurs until the remaining internal energy is depleted. This is the emission that IR telescopes observe.

The process of IR emission is modelled via a straightforward cascade emission process that has been described in detail in the literature (Cook & Saykally 1998; Pech, Joblin & Boissel 2002; Basire et al. 2011; Mackie et al. 2018). In brief, the probability of emitting an IR photon of a given wavelength is proportional to the magnitude of the vibrational frequency of the corresponding normal mode multiplied by the energy-dependent emission at the given internal energy. An IR photon derived from the anharmonic vibrational frequency calculations is chosen based on the probability described above, resulting in energy loss equal to the photon energy. In short, this process uses VPT2 to compute the anharmonicity constants via the following equation:


(2) \begin{eqnarray*}
E(n) = \sum _k \omega _k \left(n_k + \frac{1}{2}\right) + \sum _{k\le l}x_{kl} \left(n_k +\frac{1}{2}\right)\times \left(n_l +\frac{1}{2}\right),
\end{eqnarray*}

where $\omega$ represents the harmonic frequency and *n* represents the number of quanta in the vibrational mode. Transition energies between adjacent vibrational energy levels of a given mode are then calculated by


(3) \begin{eqnarray*}
\Delta E^{^{\prime }(k)}(\lbrace n\rbrace) = \omega _k + 2x_{kk}(n_k) + \frac{1}{2} \sum _{i\ne k}x_{ik} + \frac{1}{2} \sum _{i\ne k}x_{ik}n_i,
\end{eqnarray*}

where $n_k$ is relative to the upper state and the $n_i$ represents the ‘spectator’ mode that is populated but is not involved in the transition. The total remaining internal energy within the molecule is incorporated by the vibrational modes that are populated, $n_i$. At this new internal energy, the energy-dependent emission spectrum is recalculated and given a new set of emission probabilities. This is repeated until the molecule has relaxed to its vibrational ground state. A Monte Carlo approach is utilized, which repeats the entire process for different probability draws, to improve accuracy and yield the final cascade emission spectrum. The details of the cascade emission simulations have been thoroughly described and validated in the literature (Mackie et al. 2015b, 2018, 2022).

## 3 RESULTS AND DISCUSSION

### 3.1 IR absorption spectra

#### 3.1.1 Indene

Indene is an unsubstituted PAH with one sp$^3$ hybridized carbon that includes two aliphatic C–H bonds attached to the ring. To the best of our knowledge, the IR spectrum of neutral indene has not been studied previously (nor its isomer 2-ethynyltoluene), and only its microwave spectrum (Li, Jalilian & Durig 1979) and the vibronic transitions (D$_{2}$–D$_{0}$, D$_{4}$–D$_{0}$) of the cation (Chalyavi et al. 2013) have been reported. Measurements are performed at room temperature, which would induce rotational broadening of the measured vibrational transitions due to relaxation processes. Previous studies show that IR band positions in the CH stretching region are redshifted by $\cong$0.02 cm^$^{-1}$^ K^$^{-1}$^ and that this shift, measured for pyrene and coronene, is size dependent (Joblin et al. 1995). A shift of less than 4 cm^$^{-1}$^ might be induced at room temperature.

**Figure 1.**
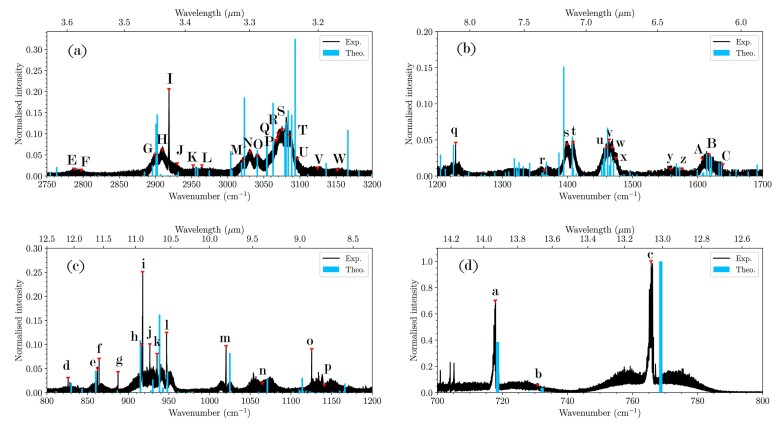
Experimental absorption spectrum (black) and anharmonic stick spectrum (blue) of indene with peak labels corresponding to assignments in Table 1. Note the different *x*-axis scale for each panel.

Fig. 1 compares the experimental and computed anharmonic spectra (blue sticks) of indene, both of which have been normalized to the maximum peak intensity. Table 1 reports the experimental band centre and relative intensity along with the anharmonic frequencies, relative intensities, and corresponding normal modes of the assigned transitions. As shown, good agreement is obtained in terms of central band positions and relative intensities which have a mean absolute error of 5.3 cm^$^{-1}$^ and 0.04, respectively, well withing the expected error range of 10–20 cm^$^{-1}$^. No peaks are detected above 3000 cm^$^{-1}$^ implying that the overtones and combination bands of the higher quanta modes do not contribute to the observed spectrum.

**Table 1.** Experimental frequencies (cm^$^{-1}$^) and relative intensities, anharmonic computational frequencies (cm^$^{-1}$^) and relative intensities, and mode assignment for Indene.

ID	Exp. frequency	Exp. intensity	Anharm. frequency	Anharm. intensity	Assignment				
a	717.8	0.70	718.5	0.39	$\nu _{36}$				
b	730.8	0.06	732.2	0.03	$\nu _{35}$				
c	765.7	1.00	768.7	1.00	$\nu _{34}$				
d	826.1	0.03	829.9	0.02	$\nu _{33}$	2$\nu _{41}$			
e	862.1	0.05	853.3	0.002	$\nu _{31}$				
f	864.2	0.07	860.1	0.05	$\nu _{32}$				
g	887.4	0.04	886.7	0.004	$\nu _{37}$ + $\nu _{45}$				
h	916.8	0.10	915.0	0.11	$\nu _{30}$				
i	917.6	0.25	919.5	0.0003	$\nu _{40}$ + $\nu _{43}$				
j	926.5	0.10	930.2	0.03	$\nu _{29}$				
k	935.4	0.08	938.4	0.16	$\nu _{28}$	$\nu _{39}$ + $\nu _{42}$			
l	947.1	0.12	948.2	0.04	$\nu _{39}$ + $\nu _{42}$	$\nu _{28}$			
m	1020.3	0.09	1024.9	0.08	$\nu _{25}$				
n	1064.9	0.02	1071.0	0.03	$\nu _{24}$				
o	1125.5	0.09	1113.9	0.03	$\nu _{22}$				
p	1141.0	0.02	1165.2	0.004	$\nu _{34}$ + $\nu _{42}$	$\nu _{26}$ + $\nu _{45}$	$\nu _{21}$		
q	1228.1	0.05	1224.8	0.043	$\nu _{18}$	$\nu _{33}$ + $\nu _{43}$			
r	1364.4	0.01	1351.7	0.001	$\nu _{29}$ + $\nu _{41}$				
			1360.7	0.005	$\nu _{26}$ + $\nu _{42}$				
			1362.9	0.006	$\nu _{27}$ + $\nu _{41}$	$\nu _{15}$			
			1365.0	0.002	$\nu _{33}$ + $\nu _{40}$	$\nu _{27}$ + $\nu _{41}$			
			1367.7	0.02	$\nu _{15}$	$\nu _{33}$ + $\nu _{40}$			
s	1399.0	0.04	1386.9	0.03	2$\nu _{37}$	$\nu _{14}$			
			1393.6	0.01	$\nu _{32}$ + $\nu _{40}$				
			1394.2	0.15	$\nu _{14}$	2$\nu _{37}$			
t	1408.6	0.04	1407.5	0.05	$\nu _{31}$ + $\nu _{39}$	$\nu _{25}$ + $\nu _{43}$			
			1409.0	0.01	$\nu _{25}$ + $\nu _{43}$				
			1413.4	0.003	$\nu _{36}$ + $\nu _{37}$				
u	1460.4	0.04	1452.5	0.01	$\nu _{32}$ + $\nu _{38}$	$\nu _{12}$			
			1455.3	0.02	$\nu _{24}$ + $\nu _{43}$	$\nu _{32}$ + $\nu _{38}$	$\nu _{12}$	$\nu _{13}$	
			1457.3	0.04	$\nu _{24}$ + $\nu _{43}$	$\nu _{12}$	$\nu _{34}$ + $\nu _{37}$	$\nu _{13}$	
v	1463.9	0.05	1464.5	0.001	2$\nu _{35}$	$\nu _{34}$ + $\nu _{37}$			
			1466.4	0.003	2$\nu _{35}$	$\nu _{34}$ + $\nu _{37}$	$\nu _{12}$		
w	1468.4	0.04	1461.7	0.07	$\nu _{13}$	$\nu _{34}$ + $\nu _{37}$			
x	1474.4	0.025	1470.8	0.002	$\nu _{30}$ + $\nu _{39}$				
y	1558.6	0.01	1558.6	0.002	$\nu _{25}$ + $\nu _{40}$	$\nu _{11}$			
z	1575.8	0.01	1572.0	0.004	$\nu _{31}$ + $\nu _{36}$				
A	1607.1	0.03	1606.7	0.002	$\nu _{24}$ + $\nu _{40}$	$\nu _{18}$ + $\nu _{43}$			
			1607.6	0.004	$\nu _{18}$ + $\nu _{43}$	$\nu _{24}$ + $\nu _{40}$	$\nu _{30}$ + $\nu _{37}$		
			1609.3	0.005	$\nu _{30}$ + $\nu _{37}$	$\nu _{18}$ + $\nu _{43}$			
B	1617.4	0.02	1614.0	0.03	$\nu _{9}$	$\nu _{31}$ + $\nu _{34}$			
			1618.5	0.03	$\nu _{31}$ + $\nu _{34}$	$\nu _{29}$ + $\nu _{37}$			
			1621.3	0.03	$\nu _{29}$ + $\nu _{37}$	$\nu _{31}$ + $\nu _{34}$	$\nu _{37}$ + $\nu _{37}$		
C	1640.4	0.01	1632.9	0.02	$\nu _{27}$ + $\nu _{37}$	$\nu _{30}$ + $\nu _{36}$			
			1636.6	0.02	$\nu _{30}$ + $\nu _{36}$	$\nu _{27}$ + $\nu _{37}$	$\nu _{29}$ + $\nu _{37}$		
D	1941.5	0.01	1937.7	0.04	2$\nu _{26}$				
E	2787.4	0.01	2754.6	0.01	$\nu _{14}$ + $\nu _{15}$	2$\nu _{14}$			
F	2797.3	0.01	2763.4	0.01	$\nu _{2}$ + $\nu _{14}$	$\nu _{14}$ + $\nu _{15}$			
G	2900.0	0.05	2884.8	0.01	$\nu _{10}$ + $\nu _{17}$	$\nu _{11}$ + $\nu _{16}$			
H	2909.4	0.06	2895.9	0.03	$\nu _{9}$ + $\nu _{17}$	$\nu _{8}$			
I	2918.8	0.20	2900.9	0.12	$\nu _{7}$				
			2902.5	0.15	$\nu _{8}$	$\nu _{9}$ + $\nu _{17}$			
J	2930.1	0.03	2931.7	0.03	$\nu _{9}$ + $\nu _{16}$	$\nu _{11}$ + $\nu _{15}$			
K	2952.3	0.02	2955.6	0.02	$\nu _{10}$ + $\nu _{15}$	$\nu _{11}$ + $\nu _{14}$			
L	2964.4	0.02	2958.5	0.02	$\nu _{11}$ + $\nu _{14}$	$\nu _{10}$ + $\nu _{15}$			
M	3021.3	0.03	3004.2	0.03	$\nu _{11}$ + $\nu _{13}$	$\nu _{6}$			
N	3030.9	0.05	3019.2	0.03	$\nu _{11}$ + $\nu _{12}$	$\nu _{11}$ + $\nu _{13}$			
			3023.2	0.19	$\nu _{6}$	$\nu _{4}$	$\nu _{9}$ + $\nu _{12}$	$\nu _{11}$ + $\nu _{13}$	
O	3041.2	0.04	3041.0	0.05	$\nu _{10}$ + $\nu _{13}$	$\nu _{5}$	$\nu _{11}$ + $\nu _{13}$		
P	3066.7	0.84	3053.9	0.10	$\nu _{5}$	$\nu _{10}$ + $\nu _{12}$	$\nu _{11}$ + $\nu _{12}$	$\nu _{4}$	
			3054.6	0.06	$\nu _{10}$ + $\nu _{12}$	$\nu _{10}$ + $\nu _{13}$	$\nu _{3}$	$\nu _{9}$ + $\nu _{13}$	$\nu _{5}$
Q	3068.8	0.09	3079.2	0.10	$\nu _{2}$ + $\nu _{14}$	$\nu _{1}$			
R	3070.0	0.10	3080.8	0.13	$\nu _{9}$ + $\nu _{13}$	$\nu _{3}$	$\nu _{2}$		
S	3075.2	0.11	3083.8	0.16	$\nu _{10}$ + $\nu _{12}$	$\nu _{3}$	$\nu _{10}$ + $\nu _{13}$	$\nu _{4}$	$\nu _{9}$ + $\nu _{13}$
			3062.8	0.17	$\nu _{3}$	$\nu _{9}$ + $\nu _{13}$	$\nu _{10}$ + $\nu _{12}$	$\nu _{9}$ + $\nu _{12}$	
T	3085.4	0.09	3093.3	0.33	$\nu _{9}$ + $\nu _{12}$	$\nu _{4}$	$\nu _{9}$ + $\nu _{13}$	$\nu _{6}$	
U	3096.7	0.03	3095.2	0.003	$\nu _{7}$ + $\nu _{45}$				
V	3124.4	0.01	3136.1	0.03	2$\nu _{11}$	$\nu _{1}$			
W	3151.5	0.01	3166.3	0.11	$\nu _{10}$ + $\nu _{11}$				

In the 3000–3100 cm^$^{-1}$^ range (Fig. 1a), a congested spectrum shows a multipeak structure resulting from complex mixed states of the aromatic C–H stretches. The theoretical spectrum reproduces the frequency positions well while only slightly overestimating the overall intensity. Theory predicts that the 3000–3100 cm^$^{-1}$^ region is governed by highly mixed vibrational states involving the highest frequency vibrational modes ($\nu _{1}$ through $\nu _{6}$) together with different combination bands. The $\nu _{3}$ symmetric in-plane aromatic CH stretch is one of the most active modes and is found to be coupled to many transitions (e.g. bands P, R, and S in Table 1). The $\nu _{6}$ in-plane stretching mode involving the two-ring system is also coupled to many transitions, e.g. M, N, J, and T. The out-of-plane aromatic CH stretch modes ($\nu _{1}$, $\nu _{2}$, $\nu _{4}$, and $\nu _{5}$) are coupled to various combination bands across the 3000–3100 cm^$^{-1}$^ region. One of the most active combination bands is $\nu _{11}+\nu _{13}$ (in-plane CC skeletal distortion and breathing), contributing to the M, N, and O bands.

The mixed aliphatic C–H stretches of indene are observed in the 2700–3000 cm^$^{-1}$^ spectral range. Here, the theoretical spectrum reproduces the intensities but the frequencies are slightly redshifted ($< $50 cm^$^{-1}$^). The strongest absorption at 2918.8 cm^$^{-1}$^ (*I*) is not resolved experimentally and, as predicted quantum chemically, is a convolution of two modes ($\nu _{7}$ and $\nu _{8}$). The features *E* and *F*, detected experimentally and predicted theoretically, each have $\nu _{14} + \nu _{15}$ character mixed with the first overtone of $\nu _{14}$ (*E*) and the $\nu _{14} + \nu _{2}$ combination band (*F*).

Several unresolved and weak features are measured in the 1200–1700 cm^$^{-1}$^ region (Fig. 1b). Absorption in this range is commonly attributed to carbon skeletal stretching. A pair of absorption structures in the vicinity of 1620 cm^$^{-1}$^ (6.2 µm) (bands *A, B*, and *C* in Table 1) and between 1400 and 1500 cm^$^{-1}$^ (*s, t, u, v, w*, and *x*) are observed here. The quantum chemical computations predict that these features arise from highly coupled overlapping overtones and combination bands. Band *s*, for example, is comprised of three coupled overlapping transitions. This includes the two coupled, opposite transitions with majority contribution from $2\nu _{37}$ and $\nu _{14}$, and a weak transition of the combination band $\nu _{32} + \nu _{40}$. Band *u* has highly mixed and coupled states where the combination band $\nu _{32}+\nu _{38}$ (the sp$^3$ C–H bending) is very active along with the $\nu _{12}$ out-of-plane C–H bend and the $\nu _{13}$ in plane C–C stretch. Similarly, in the second feature, around 1620 cm^$^{-1}$^, every identified experimental band (*A, B*, and *C*) derives from complex mixed states involving mainly combination bands.

In the 850–1200 cm^$^{-1}$^ region (Fig. 1c), the experimental and theoretical spectra are consistent. Here, the absorption features derive mainly from fundamental vibrations except for peaks *i, p*, and the two coupled states $\nu _{28}$ and $\nu _{39}+ \nu _{42}$ that result in bands *k* and *l*. Band *i* is the strongest feature in the experimentally measured spectrum while *l* is the strongest theoretically computed. Most of the vibrations in this region are in-plane CC deformations (peak *h*), in- and out-of-plane C–H bends (*k, m*, and *o*), except for the aliphatic out-of-plane C–H twist on the sp$^3$ carbon (peak *f* and *n*).

Finally, in the 700–800 cm^$^{-1}$^ region (Fig. 1d) the features *a* and *c* at 717.8 and 765.7 cm^$^{-1}$^, respectively, match the quantum chemical frequencies and intensities very well. The strongest vibration at 765.7 cm^$^{-1}$^ corresponds to the $\nu _{34}$, symmetric out-of-plane aromatic CH bending. The vibrational spectra derived from the D$_2$–D$_0$ electronic transition exhibit the same feature at 719 cm^$^{-1}$^, but the peak at 765.7 cm^$^{-1}$^ is absent. This might be due to a large change in the excited state geometry and/or a low Franck–Condon overlap between the D$_2$–D$_0$ vibrational wave functions.

#### 3.1.2 2-Ethynyltoluene

**Figure 2.**
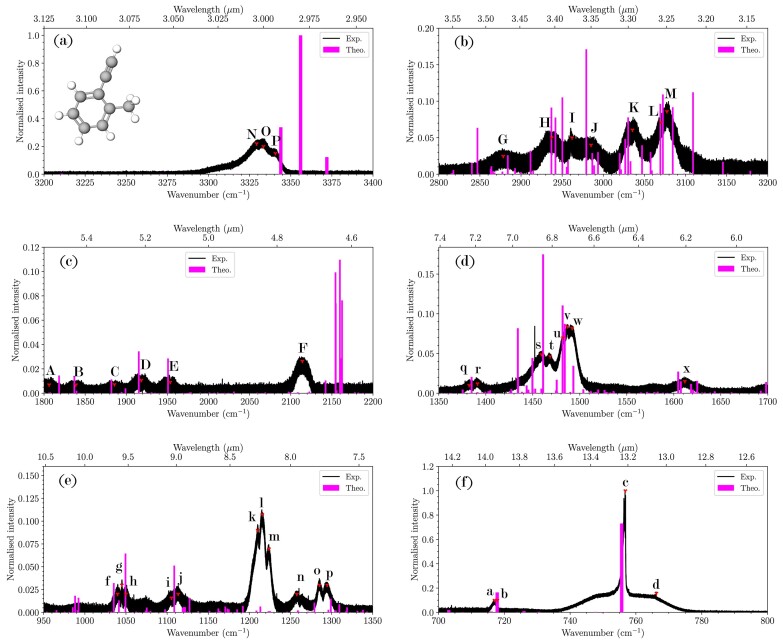
Experimental absorption spectrum (black) and anharmonic stick spectrum (pink) of 2-ethynyltoluene with peak labels corresponding to assignments in Table 2. Note the different *x*-axis scale for each panel.

Fig. 2 presents the high-resolution gas-phase laboratory absorption spectrum of 2-ethynyltoluene from 3400 to 700 cm^$^{-1}$^ and Table 2 lists the experimental band centre and relative intensity along with the anharmonic frequencies, relative intensities, and corresponding normal modes of the assigned transitions. The figure shows the computed anharmonic vibrational transitions in pink. Overall, 43 peaks are identified in the laboratory spectrum that show excellent agreement with the computations (mean absolute errors of 7.85 cm^$^{-1}$^ for the frequency and 0.03 for the relative intensities). The broad feature seen in panel (a) is composed of three overlapping peaks at 3329.2 (N), 3333.4 (O), and 3340.7 cm$^{-1}$ (P). Peaks N and O are transitions to coupled, highly mixed states with both having contributions from the ethynyl CH stretch fundamental ($\nu _1$) and the $\nu _9$ + $\nu _{20}$ combination band; however, peak O has 68 per cent $\nu _1$ character, while N has only 23 per cent. Feature P has almost complete $\nu _9$ + $\nu _{19}$ character, but draws all of its intensity from resonance coupling with $\nu _1$. The quantum chemical computations predict peaks O and P to be the first- and third-most intense peaks in the spectrum while they are seen in the experiment to have the third- and second-largest intensities. In this case, the computations overestimate the intensity of the ethynyl CH stretch, most likely due to the large dipole displacement in the normal mode being poorly described by the DFT dipole moment surface. Considering the accuracy of the anharmonic B3LYP/N07D intensities for transitions in the CC and CH bend regions herein and in previous studies (Mackie et al. 2015b, c, 2016; Esposito et al. 2024c, d, f), the error in the ethynyl CH intensity highlights a shortcoming of the B3LYP/N07D methodology. A similar result has been reported on the study of ethynylbenzene (Esposito et al. 2024d), potentially pointing to an overall issue with describing the intensities of normal modes originating from PAH side groups, such as the ethynyl group here (Finazzi et al. 2024).

**Table 2.** Experimental frequencies (cm$^{-1}$) and relative intensities, anharmonic computational frequencies (cm$^{-1}$), anharmonic relative intensities, and mode assignment for 2-ethynyltoluene.

ID	Exp. frequency	Exp. intensity	Anharm. frequency	Anharm. intensity	Assignment				
a	717.0	0.10	716.0	0.01	2$\nu _{30}$ + $\nu _{31}$				
b	717.9	0.10	717.8	0.16	$\nu _{32}$				
c	756.8	1.00	755.7	0.73	$\nu _{30}$				
d	766.2	0.20	770.5	0.01	$\nu _{33}$ + $\nu _{44}$	$\nu _{34}$ + $\nu _{43}$			
e	943.0	0.10	940.7	0.01	$\nu _{27}$	$\nu _{29}$ + $\nu _{44}$			
f	1039.8	0.02	1035.1	0.03	$\nu _{24}$				
g	1045.0	0.03	1041.4	0.01	$\nu _{34}$ + $\nu _{38}$	$\nu _{36}$ + $\nu _{38}$			
h	1050.0	0.02	1049.4	0.07	$\nu _{23}$				
i	1105.3	0.02	1108.7	0.06	$\nu _{22}$	$\nu _{30}$ + $\nu _{40}$			
j	1113.4	0.02	1127.3	0.01	$\nu _{24}$ + $\nu _{45}$				
k	1210.1	0.09	1192.1	0.01	$\nu _{20}$	$\nu _{31}$ + $\nu _{39}$			
l	1216.0	0.10	1208.4	0.01	$\nu _{30}$ + $\nu _{38}$	$\nu _{19}$			
m	1224.0	0.06	1213.4	0.01	$\nu _{19}$	$\nu _{30}$ + $\nu _{38}$			
			1223.3	0.01	$\nu _{33}$ + $\nu _{35}$				
			1225.3	0.01	$\nu _{28}$ + $\nu _{40}$				
n	1257.8	0.02	1259.0	0.01	$\nu _{32}$ + $\nu _{36}$	$\nu _{32}$ + $\nu _{34}$			
o	1285.3	0.03	1279.1	0.01	$\nu _{18}$				
p	1294.7	0.03	1299.1	0.01	$\nu _{17}$	$\nu _{27}$ + $\nu _{40}$	$\nu _{31}$ + $\nu _{35}$		
			1309.4	0.01	$\nu _{31}$ + $\nu _{35}$	$\nu _{17}$	$\nu _{27}$ + $\nu _{40}$		
q	1382.3	0.01	1375.4	0.01	$\nu _{24}$ + $\nu _{41}$				
r	1391.2	0.01	1384.9	0.01	$\nu _{16}$				
s	1459.2	0.05	1449.6	0.04	$\nu _{17}$ + $\nu _{43}$				
t	1468.6	0.04	1461.0	0.17	$\nu _{13}$				
u	1482.1	0.07	1475.4	0.02	$\nu _{30}$ + $\nu _{32}$				
			1481.7	0.11	$\nu _{12}$	$\nu _{27}$ + $\nu _{36}$			
v	1486.8	0.08	1484.0	0.09	$\nu _{27}$ + $\nu _{36}$	$\nu _{12}$	$\nu _{27}$ + $\nu _{34}$		
w	1491.8	0.08	1493.2	0.03	$\nu _{24}$ + $\nu _{38}$				
x	1611.8	0.01	1604.6	0.03	$\nu _{24}$ + $\nu _{38}$				
			1607.6	0.01	$\nu _{13}$ + $\nu _{43}$	$\nu _{10}$			
			1618.8	0.01	$\nu _{28}$ + $\nu _{30}$				
			1624.9	0.01	$\nu _{21}$ + $\nu _{39}$				
y	1698.2	0.01	1698.2	0.01	$\nu _{27}$ + $\nu _{30}$				
z	1727.2	0.01	1721.1	0.01	$\nu _{26}$ + $\nu _{30}$				
A	1806.2	0.01	1818.3	0.01	$\nu _{27}$ + $\nu _{28}$	$\nu _{26}$ + $\nu _{30}$			
B	1838.8	0.01	1836.9	0.01	$\nu _{26}$ + $\nu _{28}$				
C	1887.1	0.01	1899.1	0.01	$\nu _{24}$ + $\nu _{28}$				
D	1918.5	0.01	1915.2	0.03	$\nu _{26}$ + $\nu _{27}$				
E	1952.8	0.01	1950.8	0.03	2$\nu _{26}$				
F	2114.1	0.02	2154.4	0.10	$\nu _{11}$ + $\nu _{35}$	$\nu _{15}$ + $\nu _{31}$			
			2154.7	0.07	$\nu _{15}$ + $\nu _{31}$	$\nu _{9}$	$\nu _{11}$ + $\nu _{35}$		
			2159.8	0.11	$\nu _{9}$	$\nu _{22}$ + $\nu _{23}$	$\nu _{15}$ + $\nu _{31}$		
			2160.4	0.03	$\nu _{21}$ + $\nu _{25}$	$\nu _{9}$			
			2162.4	0.08	$\nu _{22}$ + $\nu _{23}$	$\nu _{9}$	$\nu _{21}$ + $\nu _{25}$		
G	2879.8	0.02	2883.9	0.03	$\nu _{13}$ + $\nu _{15}$	2$\nu _{14}$	2$\nu _{15}$		
H	2937.3	0.05	2936.9	0.09	$\nu _{12}$ + $\nu _{13}$	$\nu _{8}$	2$\nu _{13}$		
			2941.8	0.08	$\nu _{12}$ + $\nu _{13}$	$\nu _{8}$			
			2950.2	0.11	$\nu _{7}$				
I	2962.0	0.05	2957.1	0.02	$\nu _{11}$ + $\nu _{16}$				
J	2985.1	0.04	2979.3	0.17	$\nu _{6}$	$\nu _{10}$ + $\nu _{16}$			
			2986.6	0.03	$\nu _{11}$ + $\nu _{15}$	$\nu _{4}$	$\nu _{5}$		
			2993.6	0.03	$\nu _{10}$ + $\nu _{16}$	$\nu _{6}$			
K	3035.5	0.05	3020.0	0.03	$\nu _{11}$ + $\nu _{13}$	$\nu _{11}$ + $\nu _{15}$	$\nu _{10}$ + $\nu _{15}$		
			3026.7	0.05	$\nu _{10}$ + $\nu _{15}$	$\nu _{5}$			
			3030.2	0.08	$\nu _{11}$ + $\nu _{13}$	$\nu _{5}$	$\nu _{31}$ + $\nu _{35}$	$\nu _{10}$ + $\nu _{12}$	$\nu _{10}$ + $\nu _{15}$
			3033.1	0.01	$\nu _{7}$ + $\nu _{45}$				
L	3068.4	0.06	3057.5	0.02	$\nu _{10}$ + $\nu _{13}$				
M	3076.4	0.06	3069.4	0.10	$\nu _{4}$	$\nu _{2}$	$\nu _{5}$	$\nu _{10}$ + $\nu _{15}$	
			3072.5	0.11	$\nu _{3}$	$\nu _{4}$	$\nu _{11}$ + $\nu _{13}$	$\nu _{11}$ + $\nu _{15}$	
			3084.4	0.09	$\nu _{2}$	$\nu _{10}$ + $\nu _{13}$	$\nu _{4}$		
N	3329.2	0.22	3343.9	0.34	$\nu _{9}$ + $\nu _{20}$	$\nu _{1}$			
O	3333.4	0.20	3356.0	1.00	$\nu _{1}$	$\nu _{9}$ + $\nu _{20}$			
P	3340.7	0.15	3371.9	0.12	$\nu _{9}$ + $\nu _{19}$				

Fig. 2, panel (b) displays the absorption spectrum from 2800 to 3200 cm^$^{-1}$^; a region that includes both the aliphatic and aromatic CH stretch fundamentals. There are seven assigned peaks in this region, *G*–*L*. The computational spectrum has transitions interspersed throughout the region, but specific groups of states seem to contribute to each of the peaks. Interestingly, the transition predicted at 2950.2 cm^$^{-1}$^ that contributes to the peak H is the only one that has a single majority contribution, in this case $\nu _7$, which is the asymmetric aliphatic CH stretch fundamental of the methyl group. The remaining peaks, G–J, are mostly composed of transitions to complex mixed states involving an aliphatic or aromatic CH stretch fundamentals mixed with a different combination band, overtone, or both. In some cases, two aliphatic CH stretches contribute to a given transition. Peak I, the spectroscopically dark $\nu _{11}$ + $\nu _{16}$ combination band, derives all of its intensity from two separate aliphatic CH stretches, $\nu _6$ and $\nu _8$, illustrating the complex anharmonic coupling that occurs in this dense spectral region and the power of the resonance polyad VPT2 approach.

The peaks assigned K, L, and M in panel (b) derive their intensity from the aromatic CH stretch fundamentals. These states are extremely mixed due to strong anharmonic coupling, as can be seen in Table 2 by the states that have majority contributions from three, four, or even five normal modes. Even though only a few modes are listed for each state within each peak assignment, each individual transition can have up to 10 modes with non-negligible contribution (defined as 1 per cent or more contribution). L is assigned to just a single mode ($\nu _{10}$ + $\nu _{13}$), and like I, this combination band derives its intensity via anharmonic redistribution from modes $\nu _{2}$, $\nu _{3}$, and $\nu _{4}$, aromatic CH stretches. The state mixing across the entire spectrum, but especially in this region, shows the complexity of the vibrational structure for PAHs. This complexity is brought on from the high density of vibrational states and strong anharmonic coupling, which requires advanced theoretical treatment such as the methods used here to fully untangle the spectrum.

The remainder of the laboratory spectrum shows good agreement with the quantum chemical computations, allowing for an assignment of each peak to one or multiple transitions. Other features of note include F in panel (c), the ethynyl CC stretch measured at 2114.1 cm^$^{-1}$^, which is overestimated by the computations by approximately 40 cm^$^{-1}$^. This overestimation follows the trend also seen in the ethynyl CH stretch region mentioned above, and continues the theme of the difficulty using B3LYP/N07D to describe the ethynyl-functional group vibrational states. The ethynyl CC stretch is also overestimated in ethynylbenzene (Esposito et al. 2024d).

Similar to the indene spectrum, a pair of vibrational spectral features is observed in the 1350–1700 cm$^{-1}$ region (Fig. 2d). The first band (*x*) at 1611.8 cm^$^{-1}$^ (6.2 µm) is weaker than the indene band probably due to the lower aromaticity. The four transitions assigned to this feature are a mixture of different combination bands that include both CC skeletal motions as well as aromatic and aliphatic CH bends and wags. The 6.2 µm feature in astronomical spectra is typically attributed solely to the aromatic CC skeletal stretches, but the assigned transitions in Table 2 indicate a highly mixed aromatic/aliphatic character, similar to the other regions throughout the spectrum.

The second structure from 1450 to 1500 cm$^{-1}$ (bands *s, t, u, v*, and *w*) is stronger than indene absorption. This structure is mainly due to C–H bending modes. The quantum chemical computations predict that the strongest bands in this region are $\nu _{13}$ and the convolution of 48 per cent $\nu _{12}$ with 30 per cent $\nu _{30}+\nu _{32}$. All of these vibrations arise from CH bending in the methyl and ethynyl-functional groups, which explains the stronger intensity compared to indene. The peaks assigned *q*–*w* from about 1380 to 1495 cm$^{-1}$ include combination bands of lower frequency bending motions and fundamental transitions of different CC skeletal motions. Peak *r*, measured at 1391.2 cm^$^{-1}$^, is assigned to the $\nu _{16}$ aliphatic CH$_3$ umbrella motion of the methyl substituent, another marker of the alkyl aliphaticity of 2-ethynyltoluene compared to the ring-like aliphatic nature of indene.

Moving down to panel (e), peaks *f*–*j* from 1040 to 1115 cm^$^{-1}$^ are assigned to the various groups of computed states underneath them. Disagreement is seen for peaks *k, l*, and *m* where three overlapping features are measured in the experiment, but no major peaks are predicted by the computations. As listed in Table 2, five transitions in this region may contribute to the intensity detected here. Intensities commonly exhibit an order of magnitude error for regions of high electron density when compared with experiments, but this is unusual for the B3LYP/N07D method, considering its demonstrated accuracy (Maltseva et al. 2015; Mackie et al. 2018; Esposito et al. 2024c, d, e, f). The modes contributing to the transitions listed for features k-m are intrinsically weak, and after intensity redistribution from the anharmonic coupling in the computations, the intensity spreads thin. This is a unique phenomenon that may warrant further investigation in the future.

The broad, intense peak at 756.8 cm$^{-1}$ in panel (f) is assigned to the $\nu _{30}$ symmetric out-of-plane aromatic CH bend. The peak has a baseline width of 18 cm$^{-1}$ but does not display rotational structure like the analogous feature in indene. This is the most intense feature observed in the 2-ethynyltoluene experiment.

The lowest frequency mode, $\nu _{45}$, represents the internal methyl-rotor motion. Usually, vibrational frequency calculations and anharmonic calculations, in particular, of molecules that include a free methyl rotor are exceedingly difficult due to the extremely shallow potential energy surface along this coordinate causing unreasonable anharmonicities with QFF treatments. Fortuitously, in 2-ethynyltoluene the methyl rotor is hindered by the presence of the adjacent ethynyl group. This is exemplified by relaxed potential energy scans of the methyl rotation motion where the triply symmetric barrier is 160 cm$^{-1}$, allowing for proper characterization of its potential surface with the anharmonic methods used here. Similar computations of isomers 3- and 4-ethynyltoluene (meta and para substitutions, respectively) show a methyl rotation barrier of the order of 10 cm$^{-1}$. This shallow barrier, and subsequent difficulty computing the QFF, leads to unphysical anharmonicities in this mode of up to 10 000 cm$^{-1}$.

### 3.2 IR cascade emission spectra

**Figure 3.**
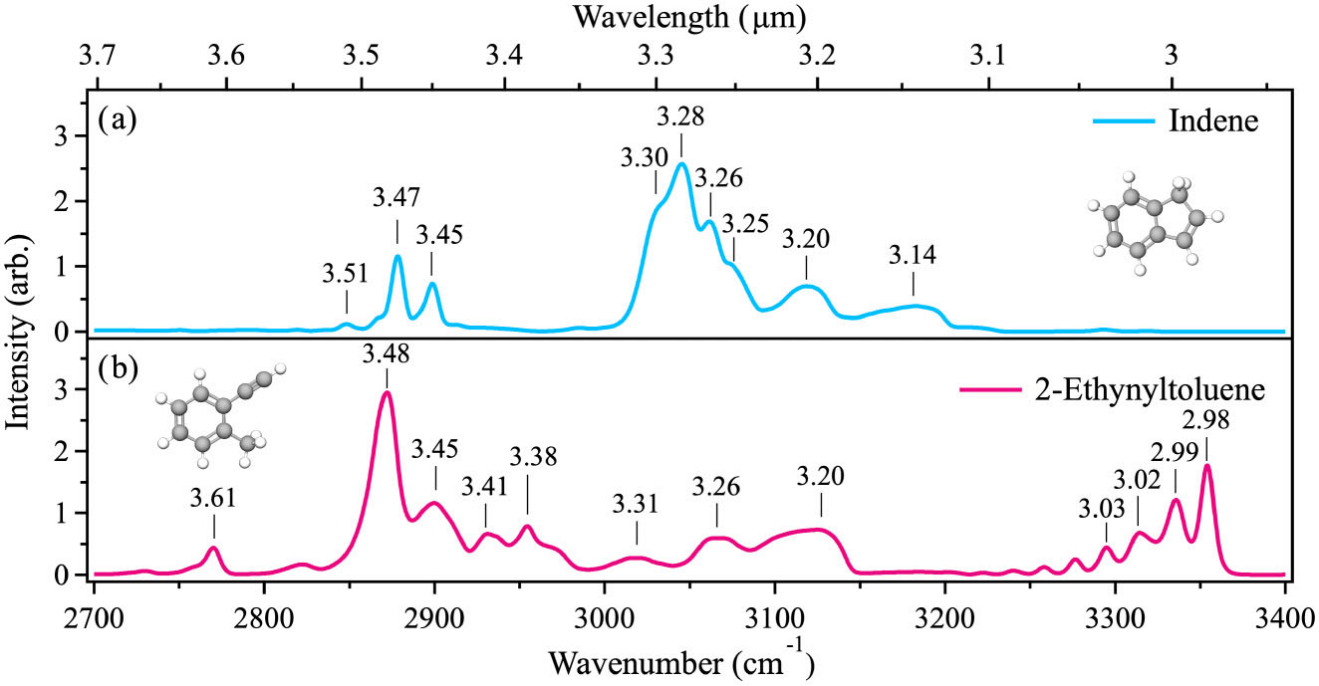
Anharmonic IR cascade emission spectra of (a) indene and (b) 2-ethynyltoluene in the CH stretch region (2700–3400 cm^$^{-1}$^; 3.7–2.9 µm) at a starting internal energy of 2 eV. Notable features are marked with their peak wavelength.

Fig. 3 presents the anharmonic IR cascade emission spectra for indene (a) and 2-ethynyltoluene (b) between 3.7 and 2.9 µm (2700–3400 cm^$^{-1}$^), encompassing the entire CH stretch fundamental regions. The simulations begin with a total internal energy of 2 eV. This energy is chosen to provide higher resolution for each feature as broadening increases with increasing internal energy (Mackie et al. 2018, 2022; Esposito et al. 2024d). Due to its direct applicability to observational astronomy, the discussion in this section will be mainly done in µm.

#### 3.2.1 Indene CH stretches

The indene spectrum shown in panel (a) of Fig. 3 is marked by two intense regions, one originating from the aliphatic and the other from aromatic CH stretches. The intense aromatic CH stretch feature from 3.32 to 3.23 µm (3000–3090 cm$^{-1}$) is characterized by three shoulder peaks at 3.30, 3.26, and 3.25 µm in addition to the tallest peak at 3.28 µm. Referring to Table 1, peaks *M*–*T* are the underlying transitions that lead to this broad emission feature, which includes contributions from the aromatic CH stretch fundamental modes $\nu _1$ through $\nu _6$ as well as various combinations bands of CH stretches, CC stretches, and CH bends. Two broad, less intense features at higher energy from the main peak occur at 3.20 and 3.14 µm. The feature at 3.20 µm stems from the experimentally assigned peak *V* that is a mixture of the $\nu _{11}$ overtone and the highest frequency aromatic CH stretch $\nu _1$. The band at 3.14 µm is peak *W*, the combination band $\nu _{10}$ + $\nu _{11}$. Both high frequency features gain all of their intensity from coupling with the intense aromatic CH stretches: $\nu _1$, $\nu _3$, $\nu _4$, and $\nu _6$ for the former and $\nu _1$ and $\nu _3$ for the latter.

At this excitation energy, there is a clear separation of approximately 100 cm^$^{-1}$^ between the aromatic CH stretch feature and the less intense, less convoluted, less broad aliphatic CH stretch features at 3.51, 3.47, and 3.45 µm. These three peaks arise from the specific transitions assigned to peaks *G, H*, and *I*, in Table 1. Peak *I* has an experimental relative intensity of 0.2 and has two underlying transitions that match the experimental intensity well, namely: (1) $\nu _{7}$, and (2) $\nu _{8}$ and $\nu _{9}$ + $\nu _{17}$.

#### 3.2.2 2-Ethynyltoluene CH stretches

The anharmonic IR cascade emission spectrum of 2-ethynyltoluene with an internal energy of 2 eV is presented in Fig. 3(b). Due to the presence of aliphatic, aromatic, and ethynyl CH stretches, there are emission features throughout the entire region. Marked differences from indene are visible throughout the spectrum. In particular, the additional high intensity features between 3.5 and 3.35 µm, lower intensity in the features between 3.35 and 3.20 µm, and the ethynyl ‘ladder’ between 3200 and 3400 cm$^{-1}$.

The presence of the methyl substituent leads to a more intense, broad 3.4–3.5 µm feature as seen in the 3.48 (2870), 3.45 (2900), 3.41 (2930), and 3.38 µm (2960 cm$^{-1}$) peaks. The assigned experimental peaks G–J listed in Table 2 lead to these emission features. The state character and intensity in this region comes from a mixture of aliphatic and aromatic components. The majority of the intensity is derived from anharmonic coupling with the aliphatic methyl CH stretches, although many of the states include aromatic and carbon skeletal components. The broadness of this region arises from the strong coupling between the methyl CH stretches and intrinsically weak combination bands and overtones as well as the artificial line shape function. Some combination bands that include a traditionally aliphatic motion with a non-aliphatic mode derive their intensity from the aliphatic CH stretches, complicating the direct attribution of aliphatic nature in this region. This is exemplified by the peak at 3.38 µm ($\sim$2960 cm^$^{-1}$^; I), where the band carrier is the $\nu _{11}$ + $\nu _{16}$ combination band. This intrinsically weak combination band combines a CC skeletal stretch with the methyl umbrella motion. The presence of the CC skeletal stretch dilutes the aliphaticity, while the intensity derived from the aliphatic CH stretches increases the influence of aliphaticity.

The peaks in the 3000–3100 cm^$^{-1}$^ region that are traditionally attributed to the aromatic CH stretches show a similarly complicated situation where state character comes from both aromatic and aliphatic CH as well as carbon skeleton motions. Indeed, the lower intensity comes from the substituted methyl and ethynyl group on the molecule. This reduces the number of aromatic hydrogens, lowering the density of strong aromatic CH stretches in this region. Additionally, the presence of only a benzene-like ring in 2-ethynyltoluene limits the range of aromatic CH stretch frequencies. In indene, the CH stretches on the 5-membered ring lead to the peak at 3.14 µm.

The ‘ladder’ of peaks around 3 µm stem solely from the ethynyl CH stretch and combination bands coupled to it. This oscillating structure has previously been seen in the related ethynylbenzene molecule and arises from strong coupling between the ethynyl CH stretch and low-frequency motions. The structure of these features is seen at the higher resolution used here; however, with higher excitation energies or with multiple ethynyl-substituted molecules present, the structure of this feature might wash out to become a broad peak with a long red tail centered at 3 µm. In PAHs, emission at this wavelength can only originate from an ethynyl group, so intensity detected here would directly point to the presence of these molecules.

#### 3.2.3 The 7.25 and 6.85 µm emission features

Based on the typical *I*$_{3.4}$/*I*$_{3.3}$ intensity ratio, the median PAH aliphatic/aromatic fraction is assumed to be 5.4 per cent (Yang & Li 2023b). The two emission features at 7.25 and 6.85 µm have previously been attributed to aliphatic CH bending motions and have been used to derive a methylene aliphatic fraction of at most 10 per cent based on the *I*$_{6.85}$/*I*$_{6.2}$ ratio (Yang et al. 2016).

**Figure 4.**
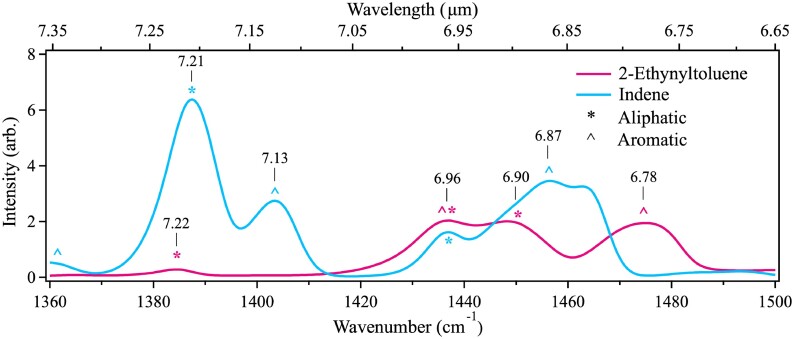
Anharmonic IR cascade emission spectra of the 7.25 and 6.85 µm emission features of indene (blue) and 2-ethynyltoluene (pink) at a starting internal energy of 2 eV. The peaks are marked with a symbol indicating the type of CH motion responsible for the feature.

Fig. 4 presents the anharmonic IR cascade spectrum of indene and 2-ethynyltoluene in the 7.25 and 6.85 µm region. The peaks are marked with symbols indicating whether the main contributor to the feature is aliphatic (asterisk) or aromatic (caret) in nature. The features at 7.13, 6.96, 6.87, and 6.78 µm in both molecules reveal major contributions from aromatic CH bending motions to the 7.25 and 6.85 µm bands. For the 7.25 µm feature in indene, aromaticity represents the smaller of the two components while in the 6.85 µm band, the aromatic constituent carries a major portion of the intensity. The integrated intensity of the indene bands at 7.21 and 7.12 µm attributes 25 per cent of the total intensity to aromaticity. The aromatic character of the 6.85 µm band of indene is much more pronounced, with an aromatic/aliphatic intensity ratio of 85 per cent/15 per cent.

The situation for 2-ethynyltoluene is slightly more complex. The small band at 7.22 µm is aliphatic in nature but with almost negligible intensity. The three broad bands at 6.96, 6.90, and 6.78 µm are highly mixed, with the first having both aliphatic and aromatic character. The bands at 6.90 and 6.78 µm are mainly aliphatic and aromatic, respectively.

The individual band structure seen in Fig. 4 is only resolved because of the low excitation energy of 2 eV used. The structure will become convoluted with higher excitation energies, making the individual aliphatic and aromatic contributions impossible to disentangle. Based on the computations in the current work, the assumption that these features stem solely from aliphatic motions is inaccurate at best. Similarly, the attribution of purely aromatic motions to the 6.2 µm feature, which is used as a marker for PAH charge fraction as well as aliphatic fraction, is challenged in this work. The misattribution of these bands in previous studies comes from use of the harmonic approximation in the quantum chemical computations. Anharmonic computations are needed to provide the most accurate representation of the spectral features. Additionally, the polyad treatment used herein gives unparalleled detail on the normal mode character of each transition, and the strong anharmonic coupling leads to highly mixed states that cannot be assigned to a single aliphatic or aromatic motion.

## 4 ASTROPHYSICAL IMPLICATIONS

**Figure 5.**
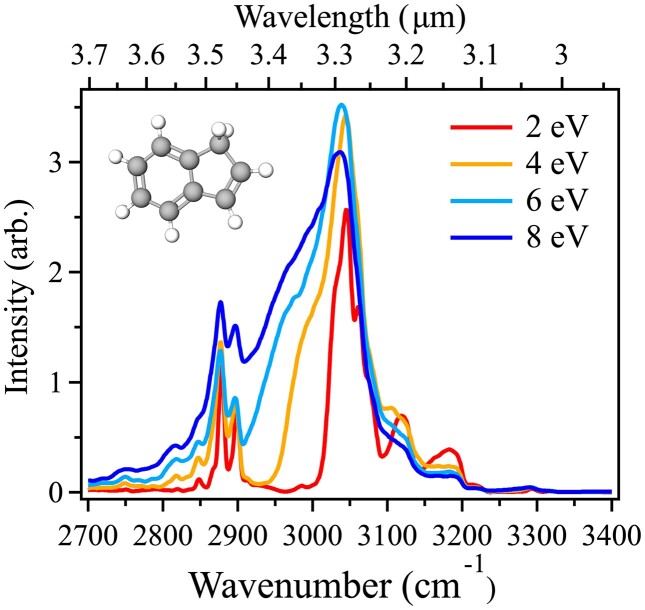
Anharmonic IR cascade emission spectra of (a) indene in the CH stretch region (2700–3400 cm^$^{-1}$^; 3.7–2.9 µm) at starting internal energies of 2, 4, 6, and 8 eV.

Astronomical PAHs are typically observed in emission after absorbing high-energy photons from their surrounding radiation field, with the smaller members of the population reaching high excitation temperatures and emitting strongly in the C–H stretching region around 3.3 µm (Ricca et al. 2012). This photoexcitation introduces considerable broadening of the red wing, which is demonstrated in Fig. 5 for indene when considering the full emission cascade at an internal energy of 2, 4, 6, and 8 eV. Notably, the red wing of the $\sim$3.3 µm feature of indene in Fig. 5 starts to blend with the feature at $\sim$3.4–3.5 µm, creating an underlying continuum that is difficult, if not impossible, to disentangle. This structure is consistent with what is seen in astronomical observations (Boersma et al. 2023; Chown et al. 2024). The difficulty in isolating the emission from these two features leads to uncertainties when attempting to quantify the aliphatic PAH fraction in astronomical sources. The $I_{3.4}/I_{3.3}$ band strength ratio is commonly used to directly probe this fraction in astronomical sources (e.g. Peeters et al. 2024). Based on the analysis of some 35 sources, Yang et al. (2017) show that $I_{3.4}/I_{3.3}< $0.25. This suggests that the AIB emitters are predominantly aromatic in nature, but likely still underestimated. Furthermore, astronomical observations indicate that the ratio is impacted by stellar effective temperature, the amount of UV radiation, and the overall starlight intensity (Yang & Li 2023b). Here, it is assumed that a reduction in the ratio is driven by the cleavage of aliphatic side groups in more intense radiation environments. This effect could be partially compensated for by the increase in the intrinsic ratio with higher excitation energies, as seen in Fig. 5. This adds additional uncertainty component to quantifying the aliphatic fraction.

Both indene and 2-ethynyltoluene have aliphatic emission structures due to the presence of one $sp^3$ carbon (Fig. 3); however, only 2-ethynyltoluene exhibits $I_{3.4}/I_{3.3}$>1. Low, likely overestimated observed $I_{3.4}/I_{3.3}$ in many astronomical sources suggests a limited presence of the methylated PAHs.

Moving now to the astronomical features at 6.85 and 7.25 µm, which are commonly ascribed to aliphatic PAH emission (Yang et al. 2016; Materese, Bregman & Sandford 2017). The data on indene and 2-ethynyltoluene presented in the current work reveal major contributions (as much as 85 per cent) from aromatic modes and even stronger blending will occur when considering excitation energies beyond 2 eV. Thus, there is a mixture of aliphatic, aromatic, and even non-CH stretch character in regions that have classically been assigned as solely aliphatic in nature (i.e. 3.4, 6.85, 7.25 µm). This complexity, revealed only by the *anharmonic* computations used here, has a direct impact on how the aliphatic fraction is determined in astronomical sources, requiring far more careful treatment.

This includes promptly revisiting the (non-) identification of features seen in IR spectra, for example the 7.24 and 7.43 µm features in JWST spectra of the Orion Bar (Chown et al. 2024). Furthermore, the mischaracterization of these bands could impact the proposed evolutionary scenario of aliphatics turning into aromatics in carbon stars with an increase in stellar effective temperature and the actual degree of inferred aromatization (Sloan et al. 2007; Acke et al. 2010). Also, the fraction of carbon atoms in aliphatics ($< $15 per cent) could actually even be less (Li & Draine 2012). Lastly, the carriers for the 3 µm plateau underlying the discrete 3.3 and 3.4 µm features would not necessarily need to be predominant aromatic in nature just because a better correlation is found with the former rather than with the latter band (Pilleri et al. 2015).

## 5 CONCLUSIONS

Various emission bands in astronomical IR spectra (e.g., 3.4, 6.85, and 7.25 µm) have been used as markers of aliphaticity in space based on harmonic vibrational frequency calculations. In reality, these peaks are actually highly mixed with both aromatic and aliphatic characters as revealed in the current study. This indicates that the amount of aliphatic carbon in space has to-date been overestimated and, therefore, has broad implications throughout astrochemistry and astrophysics.

Such conclusions have resulted from a high-level anharmonic methodology utilized to assign fully the high-resolution gas phase absorption spectra of neutral indene and 2-ethynyltoluene for the first time, allowing for the direct, simultaneous probing of the aliphatic, aromatic, and ethynyl CH stretching and bending motions. Regions with a high density of states lead to broad features consisting of highly mixed vibrational transitions in the spectra of each molecule. Further, the anharmonic IR cascade emission spectra of both molecules reveal drastic differences, such as stronger intensity from 3.55 to 3.35 µm in 2-ethynyltoluene and 3.35–3.20 µm in indene. The observed $I_{3.4}/I_{3.3}$<1 in many astronomical sources suggests a limited presence of the methylated PAHs based on the ratio obtained from the cascade emission simulations here. 2-Ethynyltoluene also has a unique band structure around 3 µm from the ethynyl substituent.

A direct connection between the microscopic world of fundamental chemistry and the macroscopic realm of astronomy has historically been difficult to make. The results herein show that there is indeed a direct need for fundamental quantum chemical and high-resolution experimental data in order to fully and correctly predict, model, and interpret macroscopic astrophysical properties. These results have begun to untangle the complex nature of the PAH IR emission bands and call for the reexamination of the aliphatic carbon fraction in the Universe.

## AUTHOR CONTRIBUTIONS

VJE - Conceptualization, data curation, formal analysis, investigation, methodology, project administration, validation, visualization, writing—original draft, writing—review and editing. SB - Conceptualization, data curation, formal analysis, investigation, project administration, validation, visualization, writing—original draft, writing—review and editing. BEB - Investigation, methodology, resources. CB - Conceptualization, writing—original draft, funding acquisition, resources, software. RCF - Methodology, supervision, writing—review and editing. FS - Funding acquisition, supervision, writing—review and editing.

## Supplementary Material

stae2588_Supplemental_File

## Data Availability

The data supporting this article have been included as part of the Supplementary Information. These include mode numbering, symmetry, and harmonic frequencies for both molecules, and rotational constants for 2-ethynyltoluene.
