# Supplementary material for: Battle of the CH motions: aliphatic versus aromatic contributions to astronomical PAH emission and exploration of the aliphatic, aromatic, and ethynyl CH stretches
Source: Mon Not R Astron Soc. 2024 Nov 15;535(4):3239–51. doi: 10.1093/mnras/stae2588 (PMC11630826; doi:10.1093/mnras/stae2588)
Supplement: stae2588_Supplemental_File [file stae2588_supplemental_file.pdf]

Supplementary Information

Battle of the CH motions: aliphatic vs. aromatic contributions to astronomical PAH emission and exploration of the aliphatic, aromatic, and ethynyl CH stretches

Vincent J. Esposito,[a†*] Salma Bejaoui,[a†] Brant E. Billingham,[b] Christiaan Boersma,[a] Ryan C. Fortenberry,[c] Farid Salama [a]

[a]*NASA Ames Research Center, MS 245-6, Moffett Field, CA 94035-1000, USA*
[b]*Canadian Light Source Inc., 44 Innovation Boulevard, Saskatoon, 11 Saskatchewan S7N 2V3, Canada*
[c]*Department of Chemistry & Biochemistry, University of Mississippi, University, MS 38677-1848, USA*

[†]These authors contributed equally to this work.

[*]Corresponding author email: vincent.j.esposito@nasa.gov

Table S1: Mode numbering, symmetry, and harmonic frequencies (cm^{-1}) for indene computed at the B3LPY/N07D level of theory.

Mode	Symmetry	Harmonic Frequency
ν_1	A′	3231.9
ν_2	A′	3208.9
ν_3	A′	3202.7
ν_4	A′	3190.8
ν_5	A′	3180.0
ν_6	A′	3173.7
ν_7	A″	3053.2
ν_8	A′	3027.1
ν_9	A′	1654.1
ν_{10}	A′	1637.8
ν_{11}	A′	1602.8
ν_{12}	A′	1492.3
ν_{13}	A′	1489.3
ν_{14}	A′	1433.6
ν_{15}	A′	1397.0
ν_{16}	A′	1351.1
ν_{17}	A′	1315.3
ν_{18}	A′	1249.6
ν_{19}	A′	1228.9
ν_{20}	A′	1184.3
ν_{21}	A′	1175.6
ν_{22}	A″	1140.7
ν_{23}	A′	1132.2
ν_{24}	A′	1087.3
ν_{25}	A′	1041.8
ν_{26}	A″	986.2
ν_{27}	A″	962.6
ν_{28}	A′	956.6
ν_{29}	A″	952.0
ν_{30}	A″	933.9
ν_{31}	A″	869.8
ν_{32}	A′	869.5
ν_{33}	A′	840.8
ν_{34}	A″	781.0
ν_{35}	A′	741.9
ν_{36}	A″	729.0

ν_{37}	A″	703.5
ν_{38}	A′	601.8
ν_{39}	A″	561.5
ν_{40}	A′	541.1
ν_{41}	A″	427.4
ν_{42}	A″	396.0
ν_{43}	A′	387.1
ν_{44}	A″	211.9
ν_{45}	A″	195.0

Table S2: Mode numbering, symmetry, and harmonic frequencies (cm^{-1}) for 2-ethynyltoluene computed at the B3LPY/N07D level of theory.

Mode	Symmetry	Harmonic Frequency
ν_1	A′	3484.3
ν_2	A′	3210.8
ν_3	A′	3200.5
ν_4	A′	3189.3
ν_5	A′	3177.7
ν_6	A′	3123.7
ν_7	A″	3094.8
ν_8	A′	3040.3
ν_9	A′	2198.2
ν_{10}	A′	1645.0
ν_{11}	A′	1609.3
ν_{12}	A′	1515.4
ν_{13}	A′	1496.0
ν_{14}	A″	1478.9
ν_{15}	A′	1466.4
ν_{16}	A′	1418.4
ν_{17}	A′	1335.0
ν_{18}	A′	1302.3
ν_{19}	A′	1236.6
ν_{20}	A′	1207.4
ν_{21}	A′	1184.4
ν_{22}	A′	1128.9
ν_{23}	A′	1067.1
ν_{24}	A″	1058.4
ν_{25}	A′	1007.4
ν_{26}	A″	992.2
ν_{27}	A″	958.4
ν_{28}	A″	879.3
ν_{29}	A′	829.2
ν_{30}	A″	771.2
ν_{31}	A′	732.9
ν_{32}	A″	727.7
ν_{33}	A′	668.3
ν_{34}	A″	623.3
ν_{35}	A′	590.0
ν_{36}	A″	582.5

ν_{37}	A′	545.7
ν_{38}	A″	469.1
ν_{39}	A′	458.1
ν_{40}	A″	387.8
ν_{41}	A′	341.4
ν_{42}	A″	217.1
ν_{43}	A′	149.3
ν_{44}	A″	132.3
ν_{45}	A″	112.7

Table S3: Rotational constants (in MHz) for 2-ethynyltoluene computed at the B3LYP/N07D level of theory.

A_0	2837.7
B_0	1472.4
C_0	975.1
Δ_J	0.414×10^{-3}
Δ_K	0.354×10^{-3}
Δ_{JK}	-0.756×10^{-3}
δ_J	0.179×10^{-3}
δ_K	-0.158×10^{-3}
ϕ_J	0.285×10^{-9}
ϕ_K	-0.178×10^{-8}
ϕ_{JK}	-0.175×10^{-8}
ϕ_{KJ}	0.324×10^{-8}
ϕ_j	0.138×10^{-9}
ϕ_k	0.104×10^{-8}
ϕ_{jk}	-0.661×10^{-9}
